# Microscopic extra-laminar sequestrectomy (MELS) for the treatment of hidden zone lumbar disc herniation: report of the surgical technique, patient selection, and clinical outcomes

**DOI:** 10.1186/s12893-021-01255-7

**Published:** 2021-05-22

**Authors:** Chunxiao Wang, Yao Zhang, Xiaojie Tang, Haifei Cao, Qinyong Song, Jiangwei Tan, Chengzhen Jin, Hongkai Song

**Affiliations:** Spine Surgery Department, Yantai Affiliated Hospital of Binzhou Medical University, Yantai, Shandong China

**Keywords:** Hidden zone, Lumbar disc herniation, Microscopic sequestrectomy, Extra-laminar approach

## Abstract

**Background:**

The area which located at the medial pedicle, posterior vertebral body and ventral hemilamina is defined as the hidden zone. Surgical management of hidden zone lumbar disc herniation (HZLDH) is technically challenging due to its difficult surgical exposure. The conventional interlaminar approach harbors the potential risk of post-surgical instability, while other approaches consist of complicated procedures with a steep learning curve and prolonged operation time.

**Objective:**

To introduce microscopic extra-laminar sequestrectomy (MELS) technique for treatment of hidden zone lumbar disc herniation and present clinical outcomes.

**Methods:**

Between Jan 2016 to Jan 2018, twenty one patients (13 males) with HZLDH were enrolled in this study. All patients underwent MELS (19 patients underwent sequestrectomy only, 2 patients underwent an additional inferior discectomy). The nerve root and fragment were visually exposed using MELS. The operation duration, blood loss, intra- and postoperative complications, and recurrences were recorded. The Visual Analog Scale (VAS), Oswestry Disability Index (ODI), and the modified MacNab criteria were used to evaluate clinical outcomes. Postoperative stability was evaluated both radiologically and clinically.

**Results:**

The mean follow-up period was 20.95 ± 2.09 (18–24) months. The mean operation time was 32.43 ± 7.19 min and the mean blood loss was 25.52 ± 5.37 ml. All patients showed complete neurological symptom relief after surgery. The VAS and ODI score were significantly improved at the final follow-up compared to those before operation (7.88 ± 0.70 vs 0.10 ± 0.30, 59.24 ± 10.83 vs 11.29 ± 3.59, respectively, p < 0.05). Seventeen patients (81%) obtained an “excellent” outcome and the remaining four (19%) patients obtained a “good” outcome based the MacNab criteria. One patient suffered reherniation at the same level one year after the initial surgery and underwent a transforaminal endoscopic discectomy. No major complications and postoperative instability were observed.

**Conclusions:**

Our observation suggest that MELS is safe and effective in the management of HZLDH. Due to its relative simplicity, it comprises a flat surgical learning curve and shorter operation duration, and overall results in reduced disturbance to lumbar stability.

## Background

Lumbar disc herniation is the most common diagnosis among degenerative disorders of the lumbar spine, affecting 2–3% of the population, and is the main cause of spinal surgery in the adult population [1–3]. Wiltse et al. [4] reported that the lateral lumbar spinal canal can be subdivided into three regions: the subarticular (lateral recess), foraminal (pedicle), and extraforaminal (far lateral) zone. MacNab [5] described the subarticular and foraminal region as the “hidden zone” due to difficult surgical exposure. Surgical management of “hidden zone” lumbar disc herniation (HZLDH) using a standard interlaminar approach requires hemilaminotomy or hemilaminectomy of the upper vertebra and is often associated with partial or complete resection of facet joints, resulting in an increased risk of lumbar segmental instability [6–9]. Di Lorenzo et al. [10] introduced a more direct approach to access the hidden zone through a fenestration on the hemilamina. Soldner et al. [11] further described this approach and named it translaminar fenestration. However, due to segment-dependent vertebral anatomy, the fenestration must be performed very laterally in the upper lumbar levels to reach the medial hidden zone. Disruption of the lateral hemilamina (pars interarticularis) has been linked to an increased risk of stress fracture and instability [12]. Dezawa et al. [13] described an endoscopic translaminar approach, in which trauma to the pars interarticularis is smaller. However this approach is more technically demanding and the surgical hand–eye coordination learning curve is steep. Therefore, while many options for the surgical management of hidden zone lumbar disc herniation exist, most of them are challenging or may result in post-operative spinal instability. In this study, we therefore developed a novel safe and effective technique called microscopic extra-laminar sequestrectomy (MELS) which we believe will be easy to implement for most surgeons. The purpose of this study is to describe this novel surgical technique, and outline patient selection and preliminary clinical outcomes used for its evaluation.

## Methods

### Patient recruitment

We undertook a non-randomized prospective study, which was approved by the ethics committee of our institution (Spine surgery department, Yantai Affiliated Hospital of Binzhou Medical University). All patients provided informed consent. Between Jan 2016 to Jan 2018, twenty one patients (13 males and 8 females) who were diagnosed with HZLDH were enrolled in this study and underwent MELS. The mean age was 59.58 ± 7.67 years, ranging from 48 to 77 years. All patients included in this study failed 4 weeks of conservative treatment before surgery. Magnetic resonance imaging (MRI) and computerized tomography (CT) were performed preoperatively and the imaging manifestations showed consistency with spinal symptoms in all patients. The inclusion criteria were as follows: (1) age > 18 years; (2) no previous history of spinal surgery; (3) MRI and CT identified the fragment was located in the hidden zone; (4) good general condition, no severe cardiopulmonary or hepatorenal dysfunction.Exclusion criteria were: (1) central stenosis (less than 10 mm) or lateral recess stenosis (less than 3 mm) confirmed by MR imaging and CT scans; (2) concomitant diseases involving systematic infection or malignant tumor; (3) segmental instability confirmed by dynamic radiographs.

### Surgical procedure

**Fig. 1:**
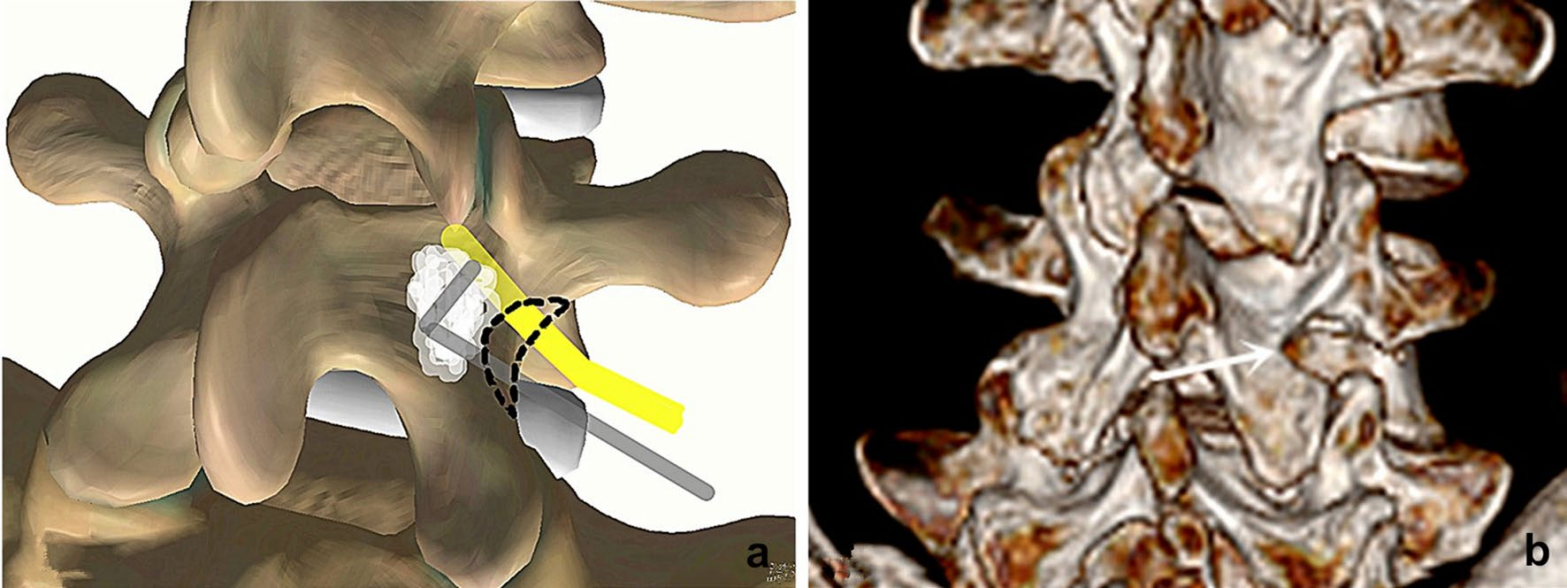
Schematic illustration of the extralaminar approach. **a** After crescent-shape excision of the lamina, the sequestered fragments can be explored with a hook along the exiting nerve root. **b** Postoperative three-dimensional reconstruction detailing the shape of excision (highlighted with the white arrow)

**Fig. 2:**
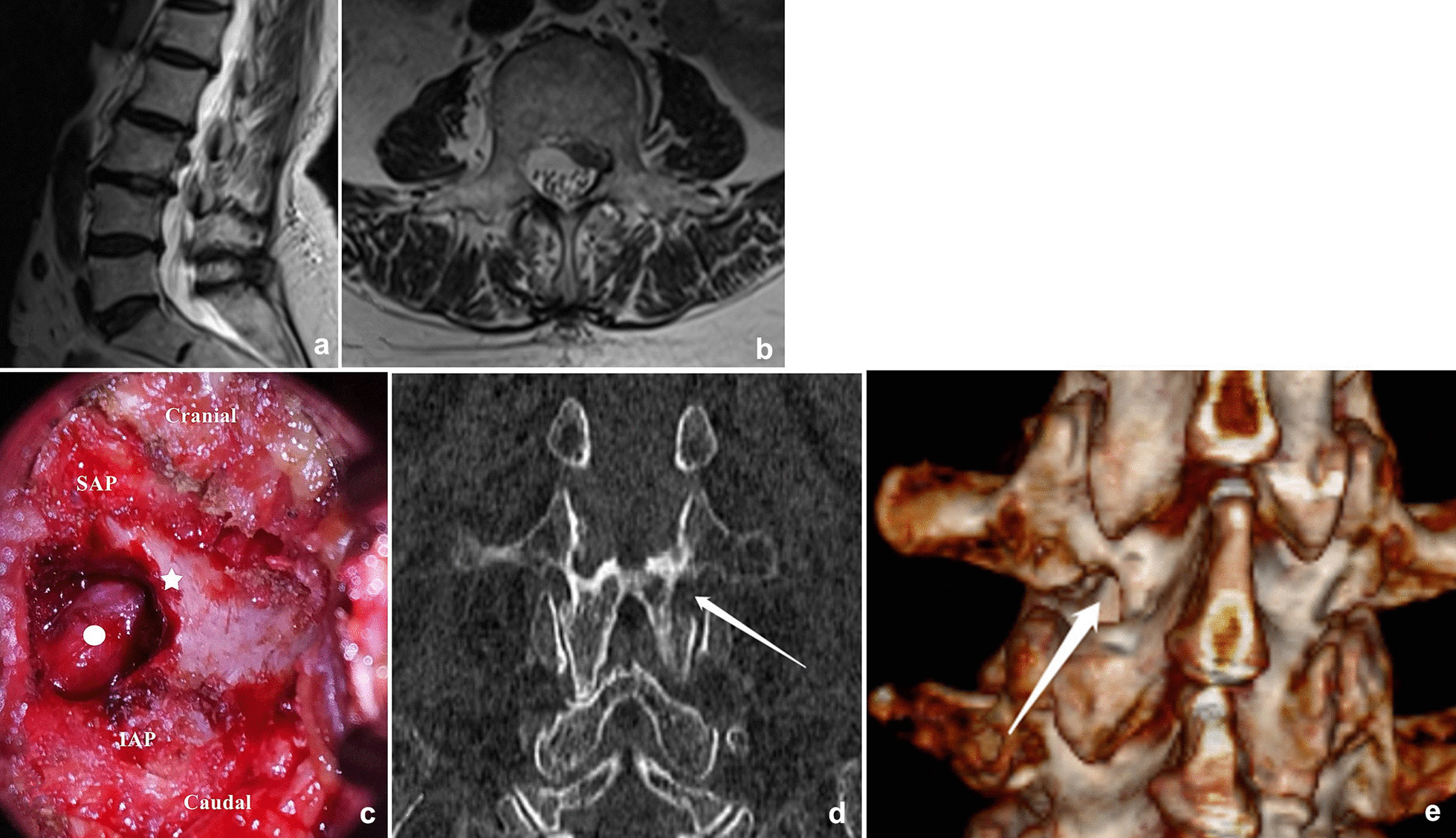
**a**, **b** The preoperative MRI showed a complete sequestered fragment. **c** The intra-operative view: the exiting nerve root was exposed after the “cresent-shaped” excision of the lamina (white circle: exiting nerve root; white star: lateral margin of the lamina; SAP: superior articular process; IAP: inferior articular process). **d**, **e** immediate postoperative CT shows the crescent-shaped excision of the lateral lamina (white arrow)

**Fig. 3:**
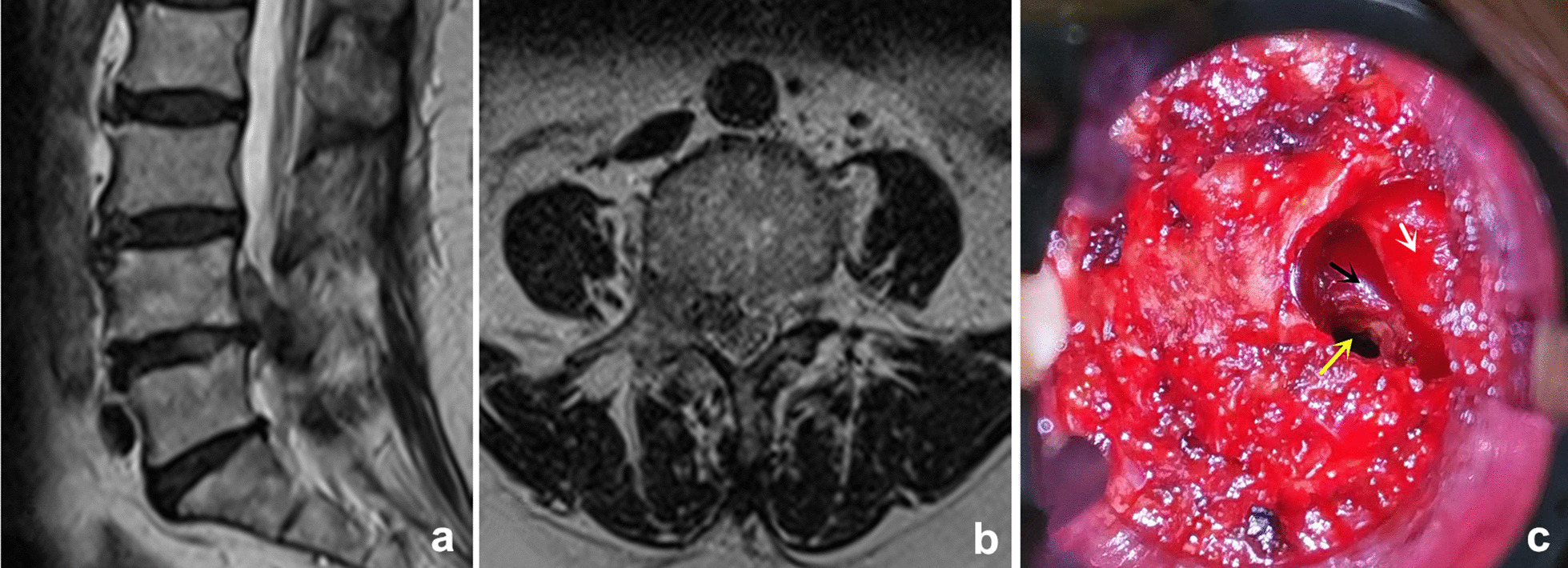
**a**, **b** The preoperative MRI showed the migrated fragments were obviously linked with the inferior disc. **c** The intervertebral space can also be approached after removing a small part of the IAP (white arrow: exiting nerve root; black arrow: posterior wall of vertebral body; yellow arrow: intervertebral space)

All the surgeries were performed by the same senior surgeon (the corresponding author). After endotracheal general anesthesia, the patient was positioned prone on the operative table, the hip and knee were flexed to widen the interlaminar space, the abdomen was kept free-hanging to reduce the intraoperative bleeding. After the spinal level of interest was identified by fluoroscopy, the Quadrant system (Medtronic, USA) was assembled and fixed to the operative table. A longitudinal incision of 2–3 cm length was made 2 cm lateral to the midline on the pathological side and a guide needle was docked on the lateral border of the lamina through the deep fascia. Sequential dilators were introduced and a suitable blade was appropriately anchored on the target lamina. After the final position of the retractor was reconfirmed by the C-arm, the lateral side of the lamina and the pars interarticularis were exposed. A blunt sublaminar dissection was performed from the lateral margin with a curette to detach the ligamentum flavum. A 2–3 mm crescent-shaped lateral lamina was excised and ligamentum flavum was removed to expose the exiting nerve root and the ganglion, around which hemostasis with a bipolar was potentially needed. This exploration was performed in a caudal to cranial direction along the nerve root with a hook probe. The nerve root was cranially retracted and appropriately protected, the sequestered nucleus pulposus was explored and removed by tracking along its path. In some cases where the routine hook probe (10 mm) was not able to reach the disc fragment, the lateral margin of the pars interarticularis would have to be removed partially to reach closer to the more medially located sequestration, while sometimes a longer hook (15 mm) was required to drag far fragments out (Fig. 1). The clearance of the intervertebral disc material depends on both preoperative MRI and intraoperative findings. The discectomy was performed in three patients as the migrated fragments were obviously linked with the inferior disc. After a small part of the superiror articular process was removed, the intervertebral space can be approached and the discectomy was easily performed (Figs. 2 and 3). After adequate hemostasis, the retractors were taken out and a latex strip drainage was placed in position before the surgical wound was sutured. The drainage was removed 24–48 h after surgery, and the stitches were 10–12 days later.

### Evaluation of outcomes

The clinical outcomes were assessed before surgery and at 3 days, 1 month, 6 months, 1 year, and 18–24 months after surgery. We used the Visual Analog Scale (VAS) to assess back and leg pain, the Oswestry Disability Index (ODI) to assess functional capacity, and the modified MacNab criteria to evaluate patient satisfaction. Operating times, blood loss, intra- and perioperative complications, the length of hospital stays and recurrences were recorded. Immediate postoperative CT and MRI images were taken for each patient to evaluate the destruction of pars and completeness of decompression. The occurrence of postoperative instability was evaluated both radiologically and clinically. A dynamic X-ray was taken at least 1 year after surgery. An anterior translation of more than 3 mm between the operated vertebra and the inferior vertebra was considered as radiological instability. New onset or significantly exacerbated back pain which induced disability was considered clinical instability.

### Statistical analysis

All data are represented as mean ± standard deviation (x ± s). The VAS and ODI before and after surgery, and at the last follow-up were compared using repeated-measures ANOVA. P < 0.05 was considered to be statistically significant. All analyses were conducted using SPSS 19.0 software (SPSS, Inc., Chicago, IL, USA).

## Results

The location of the sequestration were L2 in 2 (9.5%) patients, L3 in 8 (38.0%) patients, L4 in 9 (43.0%) patients, L5 in 2 (9.5%) patients (baseline patient characteristics are shown in Table 1). All patients had HZLDH and successfully underwent the MELS approach. The mean operation duration was 32.43 ± 7.19 min and the mean blood loss was 25.52 ± 5.37 ml. The mean length of the hospital stay was 4.19 ± 0.87 days. We observed no intra- or perioperative complications.

**Table 1:** Baseline characteristics (N = 21)

Age (range)	58.66 ± 7.77 (47–77)
Sex	
Male	13 (61.9%)
Female	8 (38.1%)
Body Mass Index (range)	25.81 ± 2.15 (22–30)
Smoking history	4 (19.0%)
Chronic comorbidity	
Hypertension	4 (19.0%)
Diabetes mellitus	2 (9.5%)
Location of sequestration	
L2	2 (9.5%)
L3	8 (38.0%)
L4	9 (43.0%)
L5	2 (9.5%)
Total	21 (100%)

### Clinical outcomes

The mean follow-up period was 20.95 ± 2.09 months (range 18–24 months). The VAS and ODI were significantly improved at all follow-up intervals (p < 0.01, Table 2). As measured using the modified MacNab criteria, 17 patients (81%) obtained an “excellent” outcome and the remaining 4 (19%) patients obtained a “good” outcome. Postoperative lower back pain occurred in 5 patients (23.8%), but could be significantly relieved with the use of painkillers. One patient suffered severe leg pain one year after the initial surgery and was diagnosed with contralateral lumbar disc re-herniation of the same level that was initially operated on. The dynamic lumbar X-Ray before the second surgery showed that no instability occurred. After performance of an endoscopic transforaminal discectomy, the patient underwent complete recovery.

**Table 2:** The changes of ODI and VAS after surgery

	Pre-op	Post-op	1 month	6 months	1 year	Last follow-up	P^1^	P^2^	P^3^
VAS^1^	7.88 ± 0.70	1.14 ± 0.79	0.43 ± 0.51	0.14 ± 0.36	0.43 ± 1.75	0.10 ± 0.30	< 0.05	< 0.05	< 0.05
VAS^2^	3.71 ± 1.38	1.62 ± 0.92	0.76 ± 0.62	0.48 ± 0.51	0.57 ± 0.93	0.19 ± 0.40	< 0.05	< 0.05	< 0.05
ODI	59.24 ± 10.83	35.81 ± 6.04	25.71 ± 7.40	14.52 ± 7.45	14.29 ± 5.63	11.29 ± 3.59	< 0.05	< 0.05	< 0.05

*Pre-op* preoperative, *Post-op* 3 days after surgery

VAS^1^ indicates the leg pain score, VAS ^2^ indicates the lower back pain score; P^1^: comparison between Pre-op and Post-op; P^2^: comparison between Post-op and 1 year; P3: comparison between Post-op and last follow-up

### Radiological outcomes

All patients showed complete removal of the fragments and no patient exhibited pars fracture as observed in the immediate postoperative CT and MRI. The dynamic X-Ray taken at least 1 year after surgery revealed that no patient showed postoperative instability.

## Discussion

In this study, we introduced MELS technique for the treatment of hidden zone disc herniation. We performed MELS on 21 patients in this study. The fragments are visually exposed and removed in all cases. Most of the patients obtained an “excellent” outcome. One patient with contralateral lumbar disc re-herniation of the same level underwent complete recovery after performing the second operation. No instability and severe back pain were observed 1 year after the surgery.

### Definition of HZLDH

**Fig. 4:**
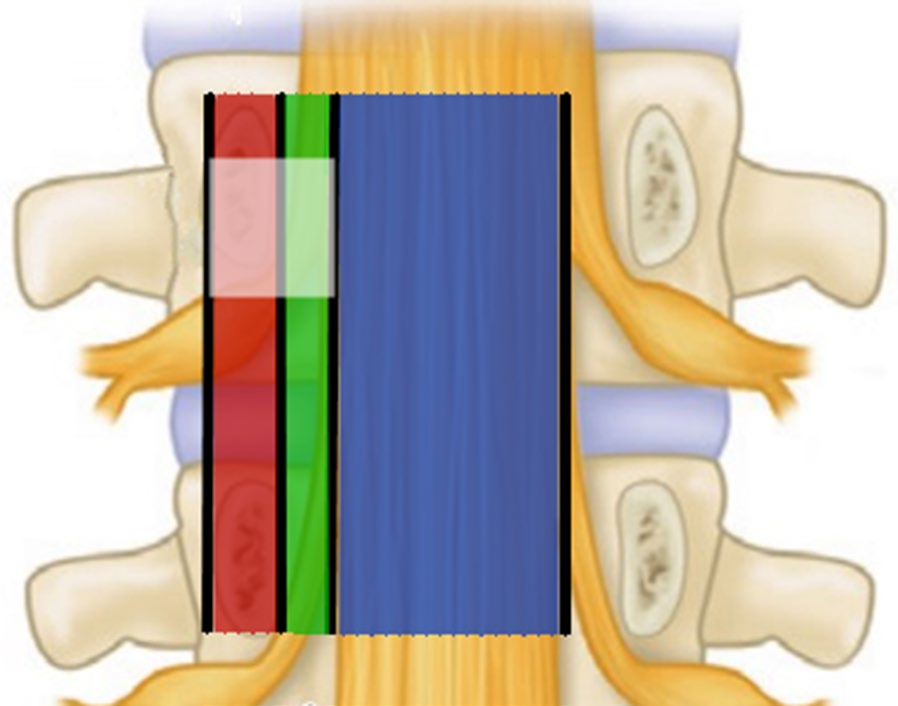
Spinal canal area classification. The red area indicates the foraminal zone (canalicular zone); the green area indicates the preforaminal zone or subarticular zone; the blue area indicates the central canal zone, and the transparent square zone indicates the hidden zone

It has been estimated that roughly 10–20% of all disc herniations migrate in a craniolateral direction and may hence be located in the preforaminal and foraminal regions of the “hidden zone” [6]. Some authors specifically defined the area which located at the medial pedicle, posterior vertebral body and ventral hemilamina as the hidden zone [5, 6]. However, the description of this type of disc herniation varies from author to author. Soldner et al. [11] use “canalicular” instead of “foraminal” and termed the HZLDH as “canalicular and cranio-posterolateral” lumbar disc herniation. Schulz et al. [14] termed sequestration in the hidden zone as “craniolateral lumbar disc herniation”. Papavero et al. [15] described this pathology as “fragment extruded cephalad into the spinal or root canal impinged the exiting root”. Despite the wide variety of descriptions, all authors mentioned above recognize the difficult surgical exposure in this clinical scenario (Fig. 4).

### Current surgical strategies

The standard surgical procedure for HZLDH is the interlaminar approach, during which removal of a major portion of the pars interarticularis is necessary [7, 9]. Donaldson et al. [16] reported that the resection of more than 50% of the facet joint is required in approximately two thirds of cases using the microsurgical interlaminar approach, which is likely to cause postoperative instability. For this reason, the interlaminar approach has gradually lost its popularity [8]. Since Di Lorenzo introduced the microscopic translaminar approach, the concept of preserving the bony borders of the lamina and sparing facet joints has been widely accepted in the management of HZLDH and has since been further described by several authors [11–14]. Several further novel approaches have been described recently. Wang et al. [17] described an approach called endoscopic transpedicle fenestration, with a bony hole drilled on the pedicle, sparing the lamina and facet joints. Reinshagen et al. [18] introduced the microscopic translaminar crossover approach. An angled fenestration is created in the contralateral hemilamina, whereby the medial portion of the hemilamina, just at the base of the spinous, is targeted to the hidden zone on the symptomatic (ipsilateral) side. This approach have superiority in recurrent patients who have surgery history of extended laminotomy. Here, we introduced the MELS for the first time to provide a novel option for the HZLDH.

### Considerations of surgical anatomy

**Fig. 5:**
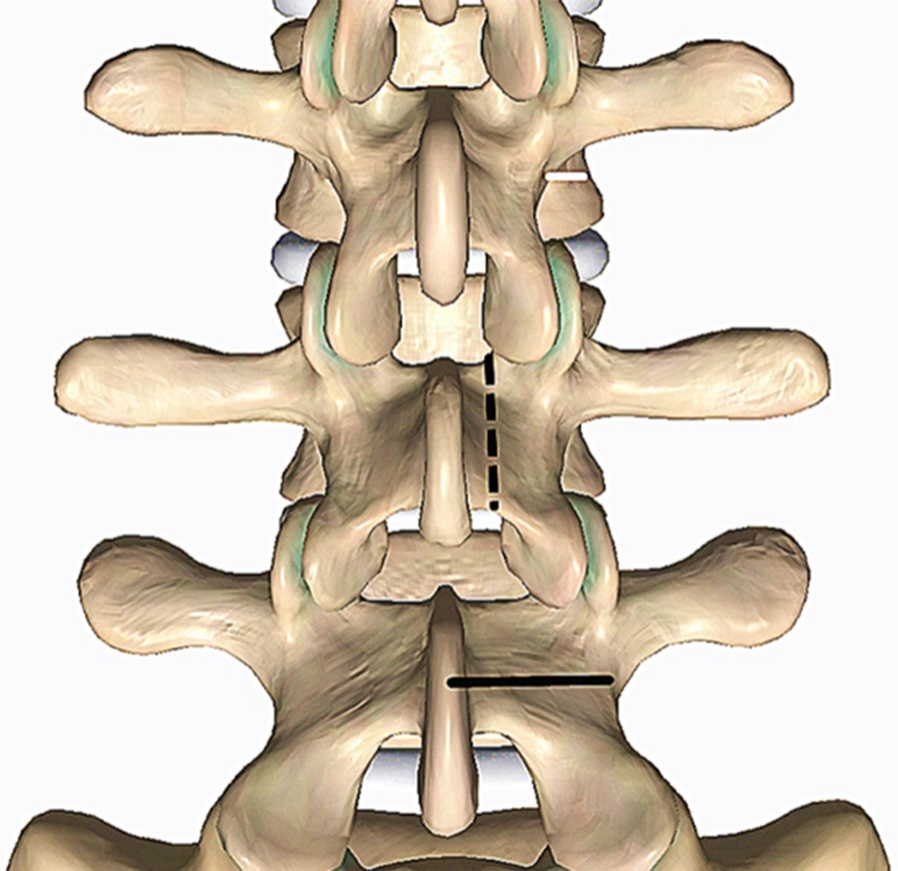
Illustration of the anatomical parameters in different levels. The solid black line indicates the width of lamina (from L5 to L3: 22 mm, 18.2 mm, 15.4 mm); the dotted black line indicates the height of lamina (from L5 to L3: 17.3 mm, 21.2 mm, 23.1 mm); the solid white line indicates the distance form lateral margin of pars to lateral border of vertebral body (from L5 to L3: 2.8 mm, 4.8 mm, 5.3 mm)

Papavero et al. [15] compared a disc fragment that is extruded cephalad underneath the lamina to a fish underneath the surface of a frozen lake, with there being two methods to hook the “fish”. The first is to cross the surface with an icebreaker and to catch the fish. The second option is to cut a small hole in the ice surface targeting the fish and to cast the rod. The translaminar approach remains popular for the treatment of HZLDH, however some authors have argued that this technique has its limitations [6]. Daghighi et al. [19] reported that disc fragments cranially migrated into the hidden zone are more commonly seen in higher lumbar levels. When performing the translaminar approach, the segment-dependent characteristic of vertebral anatomy makes the fenestration more lateral in the higher lumbar levels in order to reach the medial hidden zone [11, 15, 20] (Fig. 5). Disruption of the lateral hemilamina and pars interarticularis may increase the risk of stress fracture and instability [6, 12].

In our technique, the sequestered nuclear pulposus impinging the exiting nerve root can be compared to a melon on a vine, thus the “MELS” can be described using the Chinese saying “follow the vine to get the melon” (tracking along the stem). Along the lateral border of the lamina, the vine can be easily found, thus the melon near the vine can be explored and dragged out with a hook. When managing the higher level with relative slender lamina, an undercut of the lateral hemilamina is enough to find the nerve root and the fragment. When managing fragments at the hidden zone of the L5 level, as the above-mentioned segment-related anatomy features, the wide and short lamina of L5 lead to a relatively small operating space, thus small parts of antero-superior S1 articular process were sometimes needed to explore the deep located L5 nerve root. Ivanov et al. [12] reported that the lateral half of the pars has the largest thickness and removing one fourth of the lateral aspect of the isthmus has minimal influence on the stresses in the remaining neural arches. In the MELS, we removed only 2–3 mm lateral margin of lamina and the isthmus was maintained almost intact. No radiological instability was observed at the final follow-up in all patients and no patient suffered severe postoperative low back pain-induced disability.

### Fragmentectomy without discectomy

There is still a dispute regarding whether to deal with the intervertebral space or not in the treatment of HZLDH. Faulhauer et al. [21] proposed that fragment excision is superior to conventional disc removal due to a lower rate of postoperative spinal instability complications, while Kotil et al. [22] reported an increased recurrence rate for fragmentectomy compared with discectomy. Ebeling et al. [23] reported that cranio-lateral disc herniations commonly appear as a complete sequestration, thus management of intervertebral space is seldom needed. Moreover, Barth et al. [24] reported that discectomy did not result in additional benefits in the treatment of lumbar disc herniation, and the sequestrectomy demonstrated significantly less postoperative disc degeneration than standard microdiscectomy. In view of these considerations, we chose not to manage the intervertebral space in most of our patients. However, we performed disc removal in two cases because we found that the migrated fragments connected to the inferior disc closely, which indicated a tendency that more fragments would come out along the path. The recurrence rate of 4.8% (1/21) within the 2 years follow-up was relatively low compared with the study by Papavero et al. (7%) and was comparable with the study by Soldner et al. (3.3%) ([11, 15], respectively). However, larger patient groups and longer follow-up periods are needed to clarify the necessity of discectomy after fragmentectomy.

## Limitations

There are several limitations in this study. Firstly, our sample size was small, involving only 21 patients. Although the main purpose of this study was to introduce this novel approach, and a satisfactory outcome was obtained in the present study, larger sample sizes will be needed to provides stronger evidence for our conclusions. Secondly, management of L5 may be slightly more challenging, as the operation space is relative small. Finally, it is difficult to access the superior intervertebral space via this extralaminar approach, though the inferior discectomy is feasible. However, if superior discectomy is required, we would recommend the use of other approaches.

## Conclusion

Our results suggest that MELS is a safe and effective surgical treatment of HZLDH. This approach possesses several advantages: (1) easy to grasp for most spine surgeons, with a flat learning curve; (2) simple approach for sequestration and more reliable decompression; and (3) less disruption to the pars interarticularis and less risk of iatrogenic lumbar instability. Thus, we conclude that MELS represents a good surgical option for the treatment of HZLDH.

## Data Availability

All data used by or generated in this study is available from the corresponding author upon reasonable request.

## Abbreviations

MELS: Microscopic extra-laminar sequestrectomy
HZLDH: "Hidden zone" lumbar disc herniation
VAS: Visual analog scale
ODI: Oswestry disability index
MRI: Magnetic resonance imaging
CT: Computerized tomography
